# Visibility Graph Analysis of Reservoir-Triggered Seismicity: The Case of Song Tranh 2 Hydropower, Vietnam

**DOI:** 10.3390/e24111620

**Published:** 2022-11-07

**Authors:** Luciano Telesca, Anh Tuan Thai, Michele Lovallo, Dinh Trong Cao

**Affiliations:** 1 Institute of Methodologies for Environmental Analysis, National Research Council, 85050 Tito, Italy; 2 Institute of Geophysics, Vietnam Academy of Science and Technology, Hanoi 100000, Vietnam; tatuan@igp.vast.vn (A.T.T.); trongcd3284@gmail.com (D.T.C.); 3 Agenzia Regionale per la Protezione dell’Ambiente della Basilicata (ARPAB), 85100 Potenza, Italy; michele.lovallo@arpab.it

**Keywords:** visibility graph, reservoir-triggered seismicity, *b*-value, *k–M* slope

## Abstract

In this study, the visibility graph analysis of seismicity triggered by Song Tranh 2 hydropower (Vietnam) is performed. The relationship between the seismic (the Gutenberg–Richter *b*-value) and topological (the *k*–*M* slope) parameters of seismicity is analysed. Our findings indicate that the relationship between the Gutenberg–Richter *b*-value and the *k*–*M* slope of the investigated seismicity is in agreement with that characterising the tectonic seismicity. The results obtained from analysing the reservoir-triggered seismicity of Song Tranh 2 area could contribute to better characterisation of the relationship between the seismological and topological parameters of seismicity, strengthening the universal character of the relationship between the *b*-value and the *k–M* slope.

## 1. Introduction

Visibility graph (VG) analysis has become increasingly more employed as a statistical method to characterize the time-dynamical properties of series in various research areas, from economics [1,2,3,4] to meteorology [5,6,7], robotics and path planning [8] to medicine [9,10], and geophysics [11] to oceanography [12,13], etc.

The VG method, first developed by Lacasa et al. [14], converts time series into graphs, for which the nodes represent the series’ values, and the links or connections among the nodes represent the relationships between them. In the VG method, the relationship that define the connections among the nodes is given by the reciprocal “visibility” among the series’ values.

In recent years, the VG method has been used for the statistical investigation of seismicity; in fact, since VG is used to describe and characterize complex systems, it represents a very useful tool to disclose the complex features of seismic processes. In contrast, the ever-increasing amount of available seismicity data has permitted a deeper focus on the presence of dynamical patterns in the time distribution of seismic sequences by using a large variety of statistical methods, among which the VG represents a very powerful one.

A series of earthquakes can be viewed as a discrete time series, and the magnitudes with their own occurrence times can be considered as its values. Thus, applying the VG to this series, the magnitude time series is transformed into a graph. A graph features a variety of parameters, but one of the most important is the connectivity degree, which is the number of links connecting one node to the others on the same graph.

Telesca and Lovallo [15] first applied the VG method to Italian seismicity from 2005 to 2010; they found a collapsing effect of the distributions of the connectivity degrees of the seismic series with increasing threshold, suggesting that the properties of the connectivity degree distribution may be independent of the threshold magnitude. In the investigation of five Mexican seismic sequences, corresponding to five different tectonic areas of the subduction zone, Telesca et al. [11] found an empirical link between the *b*-value of the Gutenberg–Richter law [16] and the slope of the line fitted by a least-squares method to the relationship *k~M*; they called this the *k*–*M* slope.

Several further studies have been performed on the relationship between the *b*-value and the *k–M* slope, such as those focused on the shallow and deep Pannonian seismicities [17], in three dominant seismic areas of Northern Iran (Azerbaijan, Alborz, and Kopeh Dagh) [18], in 24 seismogenic zones in Alaska and Aleutian subduction zone at three depth ranges [19], in Taiwan and Italy [20]. Even laboratory seismicity, such as that generated by a mechanical stick-slip system with different roughness degrees [21], and physics-based synthetic seismicity [22] have shown that the *b*-value is closely linked to the *k–M* slope, suggesting that this relationship could be characterized by a type of universality.

In all the cited studies, the investigation of the relationship between the seismological and topological properties of seismicity has focused mainly on tectonic or natural seismicity. In this paper, instead, we apply the VG method to the time dynamics of the reservoir-triggered seismicity occurring around Song Tranh 2 hydropower, Vietnam. Reservoir-triggered seismicity has different generation mechanisms with respect to the tectonic seismicity; thus, our objective is to verify if the supposed universal relationship between the *b*-value and the *k–M* slope is also retained for this type of seismicity.

## 2. Seismic and Tectonic Settings

**Figure 1:**
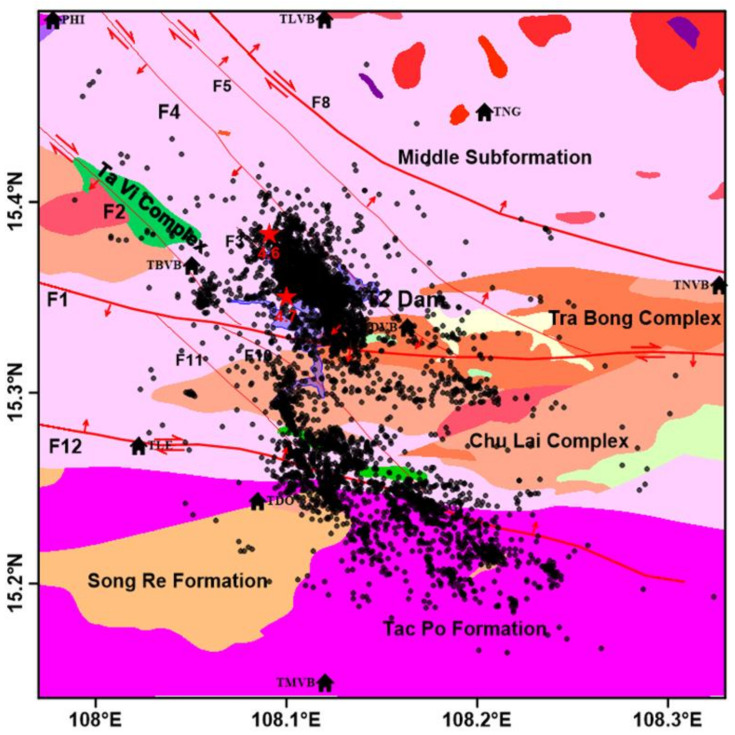
Spatial distribution of earthquake epicentres during the period of September 2012 to March 2020 (dark circles) and faults on the background of geology given by Hoai et al. [25]. The seismic stations are indicated by building symbols. The earthquakes with ML 4.7 and 4.6 are indicated by red stars.

The Song Tranh 2 hydropower and its vicinity are located on the Eastern edge of the Indochina block, close to the Western boundary of the East Sea (or South China Sea) block [23] (Figure 1). In such a position, the neo-tectonic movement takes place in the transitional area from uplift in the West to gradually falling apart in the East. Therefore, the uplift amplitude in the neo-tectonic movement tends to decrease gradually from West to East; many places in the coastal zone also show the characteristics of the subsidence zone in the Pliocene and Quaternary. Due to the influence of the collision between the Indo-Australian plate and the Eurasian plate, the lithosphere in the Song Tranh 2 region has undergone heterogeneous compression and tension movements, causing the ancient deep fault zones to reactivate during the neo-tectonic period. Studies on tectonic stress fields so far have identified that: in the neo-tectonic period, the Song Tranh 2 region has experience the influence of two tectonic phases characterized by the general movement mechanism, the Paleogene–Miocene phase has a sub-latitude direction, and the Pliocene phase up to now has a sub-meridian direction. Under the influence of the shear tectonic stress field by horizontal compression axis of the meridian and sub-meridian, the NW–SE faults have the mechanism of right-lateral strike slip motion, NE–SW faults have the mechanism of left-lateral strike slip motion [24,25]. Earthquakes started occurring in the vicinity of the Song Tranh 2 hydropower since the reservoir was impounded in 2010; before that, no earthquake had been reported in the region. The earthquakes occurred within a radius of 24 km from the centre of the reservoir. The largest earthquake of magnitudes M4.7 occurred on 15 November 2012, which caused the cracking of the houses in the Tra My district [26].

## 3. The Seismic Catalogue

Before 2012, there were no seismic stations in the Song Tranh 2 region, while in the Central region there were only a few national seismic stations located in Thua Thien Hue, Binh Dinh, Nha Trang, Da Lat, Vinh and Con Cuong. These stations are located about 130–360 km from the Song Tranh 2 hydropower. After the largest earthquake of M_L_ = 4.7 occurred in November 2012, a seismic network of ten seismic stations equipped with Guralp CMG-6TD (30 s) long-period seismometers was set up by the Institute of Geophysics—Vietnam Academy of Science and Technology (IGP-VAST). A system based on SeisComP software was used for online retrieval of seismic data. The SEISAN software of Havskov and Ottemoeller [27] was applied to analyse earthquake data using the 1D velocity model given by Son [28]. The waveforms of the particular earthquake from all the stations were analysed together to determine the hypocentral parameters i.e., the origin time, location, focal depth and magnitude. The double difference (HypoDD) algorithm of Waldhauser and Ellsworth [29] was then used to relocate earthquakes. The accuracy of the locations in horizontal parameters was between 50 and 100 m; the error in focal depth estimation was a few kilometres [30]. The seismic network allowed collection of thousands of earthquakes; during the period of September 2012 to March 2020, about 8000 earthquakes were detected with a magnitude down to −0.6.

In this study we applied the VG method not only to the whole seismic catalogue Song Tranh 2 area, but also to the declustered one.

The declustering method adopted here was developed by Zaliapin et al. [31] that separates background from clustered seismicity using the nearest-neighbour (NN) distance in the space–time–energy domain between events *i* and *j*, defined as follows [32]:

(1) $$\eta_{ij}=\left\{\begin{array}{ll} c\tau_{ij}r_{ij}^{d_{f}}10^{-wm_{i}} & \tau_{ij}>0\\ \infty & \tau_{ij}\leq 0 \end{array}\right.$$

where *τ_ij_* is the interevent time (in years) between events *i* and *j* (that is positive if earthquake *i* precede earthquake *j* or negative vice versa), *r_ij_* is their spatial distance (in km), *d_f_* is the fractal dimension of the spatial distribution of the entire seismic dataset [31], and *w* is the parameter that introduces the exponential weight of the earlier event *i* by its magnitude [33].

In this study, we set *w* equal to 0 for de-clustering purposes [34]. For unclustered or Poissonian seismicity, *η_ij_* is unimodally distributed; for clustered seismicity, *η_ij_* is bimodally distributed. Fitting the distribution of *η_ij_* with a two-component 1-D Gaussian mixture model and estimating the boundary between the two modes by the maximum likelihood method, a threshold *η*_0_ can be found, and the background and clustered seismicities can be discriminated [31].

**Figure 2:**
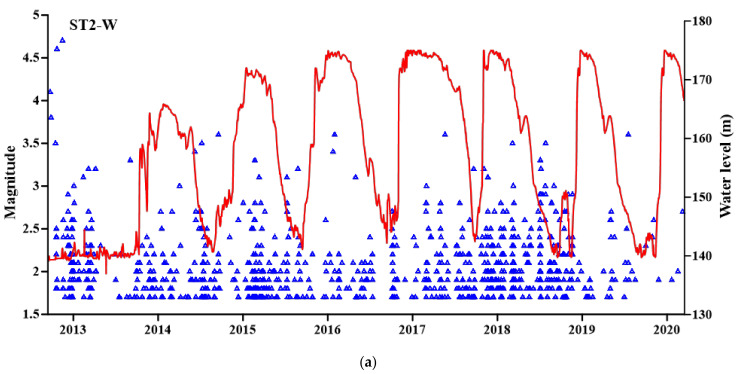

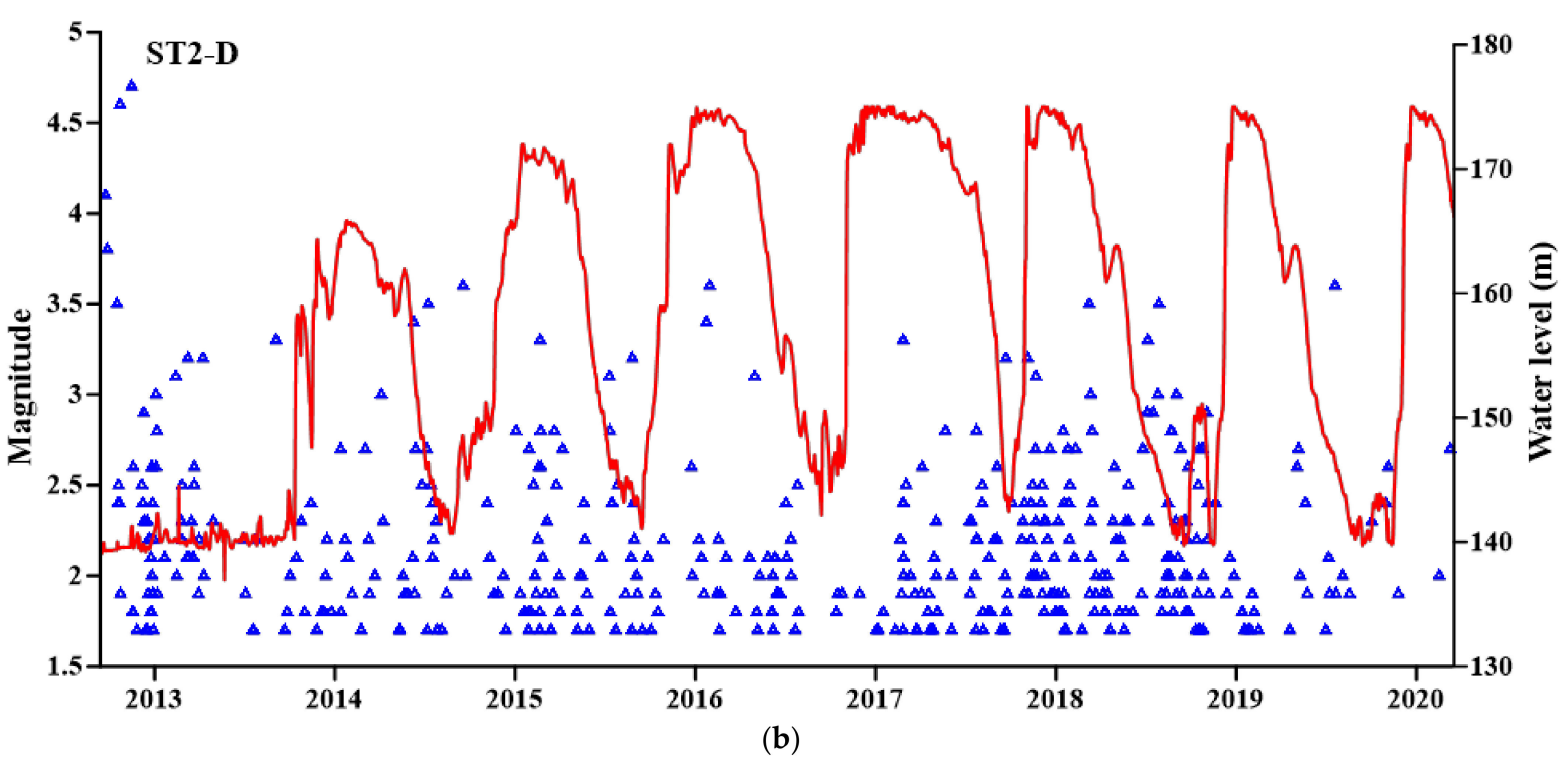
Time distribution of whole (**a**) and declustered (**b**) ST2 seismicity (blue triangles). The water level of the reservoir is indicated by the red line.

In this study, we investigated the seismicity that occurred around Song Tranh 2 reservoir only the earthquakes with a magnitude larger or equal to 1.7, which, according to Telesca et al. [35], is the completeness magnitude of whole seismicity of the area (ST-W). Figure 2a shows the time distribution of ST2-W seismicity. The application of the declustering method by Zaliapin et al. [31], produced a declustered catalogue (ST-D), for which the time distribution is shown in Figure 2b.

## 4. The Gutenberg–Richter Law

Indicated by *M_th_* the threshold magnitude and *N* the number of seismic events with magnitude *M* ≥ *M_th_*, the Gutenberg–Richter law [16] is the empirical relationship log_10_*(N)* = *a*-*bM_th_*, where *a* is the seismic productivity and *b* the proportion of smaller over larger events. The *b*-value indirectly measures the stress of a seismic area [36]. Generally, the *b*-value of reservoir-triggered seismicity is larger than that associated with tectonic earthquakes [37]. The estimation of the *b*-value can be performed by using several techniques; in this study, we employ the maximum likelihood method [38]:

(2) b=log10(e)<M>−Mc−ΔMbin2

where <*M*> is the mean magnitude of earthquake dataset with magnitude larger or equal to that of completeness *M_c_*, while Δ*M_bin_* is the width of the binning of the catalogue [39], which is typically 0.1. The error in *b* can be calculated by means of Shi and Bolt’s formula [40]:

(3) $$\sigma_{b}=2.3b^{2}\sqrt{\frac{\sum_{i=1}^{N}{\left(M_{i}-<M>\right)}^{2}}{N\left(N-1\right)}}$$

**Figure 3:**
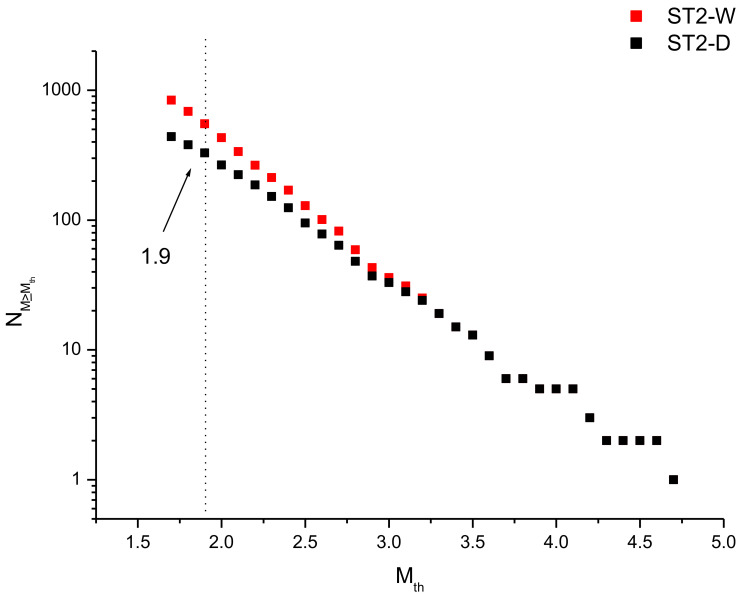
Cumulative frequency–magnitude distribution of the whole (red) and declustered (black) ST2 catalogue.

Figure 3 shows the cumulative frequency–magnitude distribution of the whole and declustered catalogues. Since when applying the method of maximum curvature [41] the completeness magnitude of ST-D is *M_c_* = 1.9, for both catalogues we considered only the earthquakes with magnitude *M* ≥ 1.9.

By using Formulae (2) and (3), the *b*-value is 0.99 ± 0.03 and 0.88 ± 0.05 for ST-W and ST-D, respectively.

## 5. The Visibility Graph Method

The VG method has been developed by Lacasa et al. [14] and transforms a time series into a graph or network, where the values of the series are converted into the nodes of the graph; the link between two nodes of the graph is established by the fulfilment of a geometrical law of “visibility”: two values of the time series *M_a_(t_a_)* and *M_b_(t_b_)* are reciprocally visible if any other value *M_c_(t_c_)* placed between them satisfies the following constraint:

(4) $$M_{c}<M_{b}+\left(M_{a}-M_{b}\right)\frac{t_{b}-t_{c}}{t_{b}-t_{a}}$$

**Figure 4:**
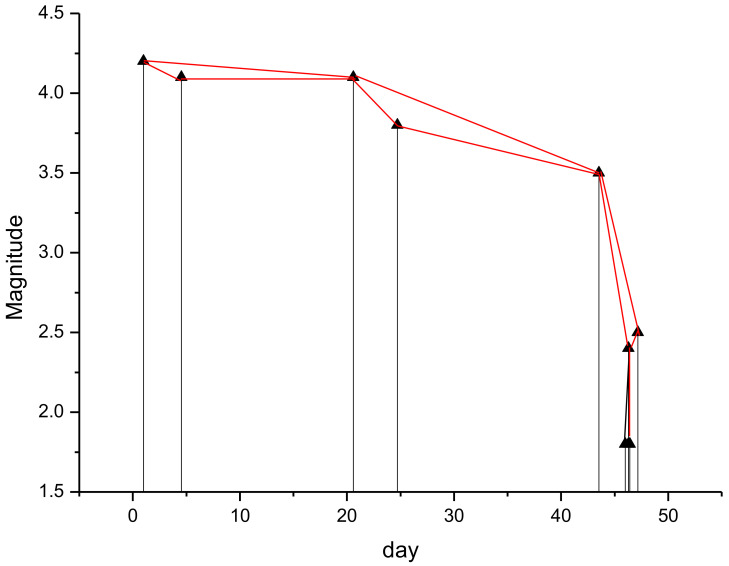
Sketch of the undirected VG method applied to the first 10 events of ST2-W seismicity. The red segments represent the connections among the events.

Thus, two values (converted into the nodes of the graph) are connected if a segment linking them is not broken by any other intermediate value. The graph built by the VG method is characterised by the following properties: (1) connectivity (each node is linked at least to its nearest neighbours (left and right), except the first and the last value of the series that are linked only to the right and left neighbours, respectively); (2) invariance under affine transformations of the time series (rescaling of both axes and horizontal and vertical translations); (3) directionality (the link could have or not direction) [14]. VG transforms periodic, random and fractal time series into regular, random and scale-free networks, respectively [42]. Figure 4 shows as an example the graph obtained considering only the first 10 earthquakes of the whole investigated seismic catalogue.

Directed visibility graphs are obtained by applying the same visibility rule of Equation (4), but connecting the values of the series only with the following (forward visibility graph) or preceding (backward visibility graph) ones.

## 6. Results

**Figure 5:**
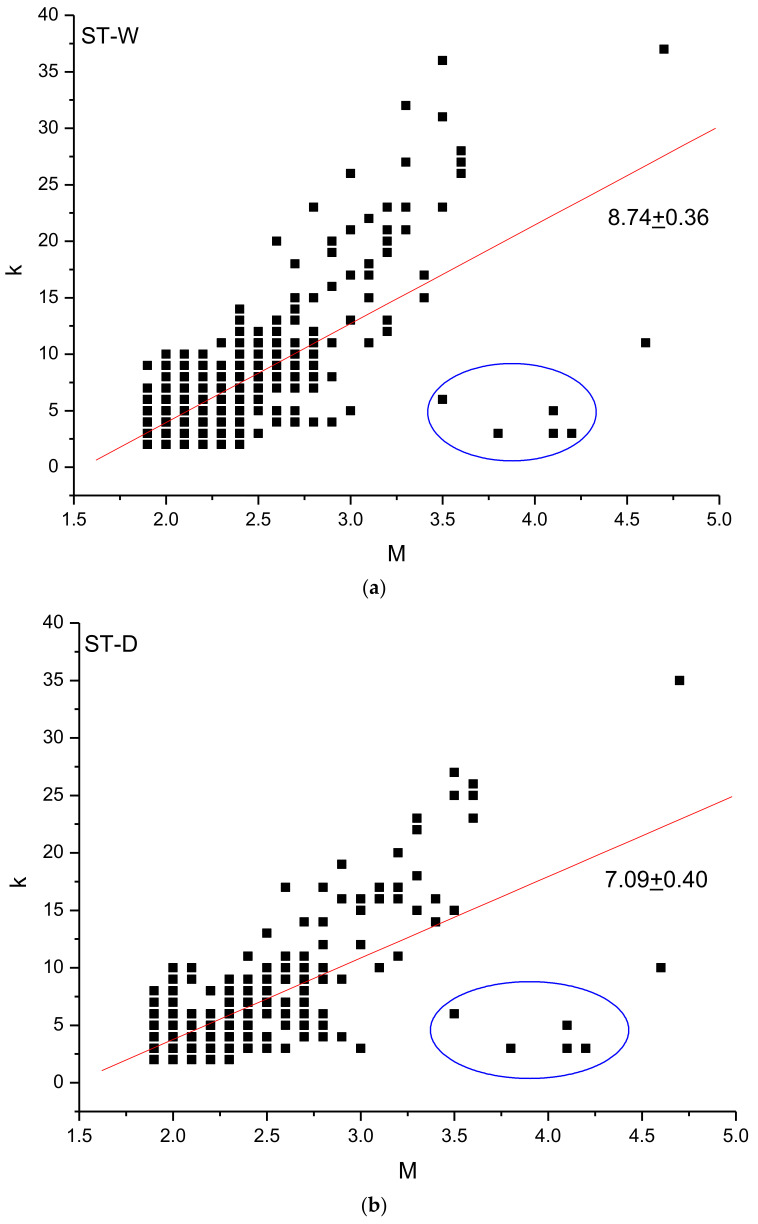

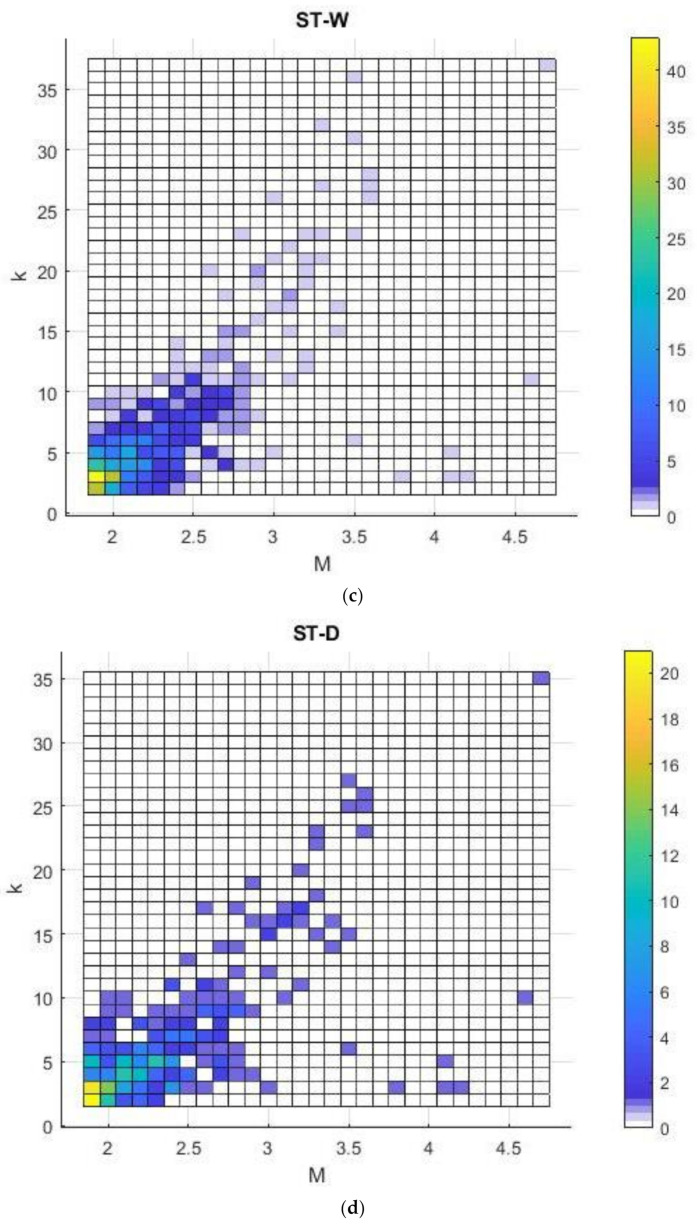
Relationship between the connectivity degree *k* and the magnitude *M* for the whole (**a**) and declustered (**b**) ST2 seismicity. The points within the blue ellipse correspond to the first five events of both catalogues (see Discussion for details). Distribution of the (*k, M*) couples for the whole (**c**) and declustered (**d**) catalogues.

Indicated by *k_i_* the connectivity degree, which is simply the number of links of node *i* (the magnitude *M_i_* in our case), we can plot *k_i_* versus *M_i_*. Figure 5 shows the *k–M* relationship for the whole (Figure 5a) and declustered (Figure 5b) catalogues, respectively. Figure 5c,d shows the number of the couples (*k, M*); as can be seen, there are many small events with the same connectivity degree *k*.

The slope of the regression line fitting the *k–M* plot is called the *k–M* slope. The positive value of the *k–M* slope indicates a positive correlation between the connectivity degree and the magnitude; thus, the larger the magnitude, the higher the connectivity degree. In fact, the stronger events with the larger magnitude act like “hubs” of the seismic time series, “attracting” more links than the smaller events; thus, they are visible by more earthquakes than the smaller ones. The *k–M* slope of the whole catalogue is 8.74 ± 0.36 while that of the declustered one is 7.09 ± 0.40. The *k–M* slope of the declustered catalogue is lower than that of the whole one; this indicates that events with the same magnitude in the whole and declustered catalogues have different connectivity degrees. Since the declustering removes clusters in the seismic sequence, which are mainly aftershocks that are close in time to the largest shocks, their removal significantly reduces the number of events that are “immediately visible” to the largest events. leading to an overall lowering of the *k–M* slope of the sequence.

Following Azzizzadeh and Cramer [19] and Telesca et al. [20], we analysed the relationship between the *b*-value and the *k–M* slope by applying the directed visibility graph (forward and backward). Telesca et al. [20] used the ratio and the difference between the two directed *k–M* slopes to characterize the “topological” isotropy of the seismicity. They found that these two measures can reveal the role played by the aftershocks, which could strengthen the connectivity in one direction rather in the other direction; after their removal, the earthquake network becomes more “isotropic” and no significant difference emerges if the events are only connected with those that have occurred before or only with those that have occurred after [20]. Such “topological isotropy” could indicate also that the events are distributed on time more homogeneously.

**Figure 6:**
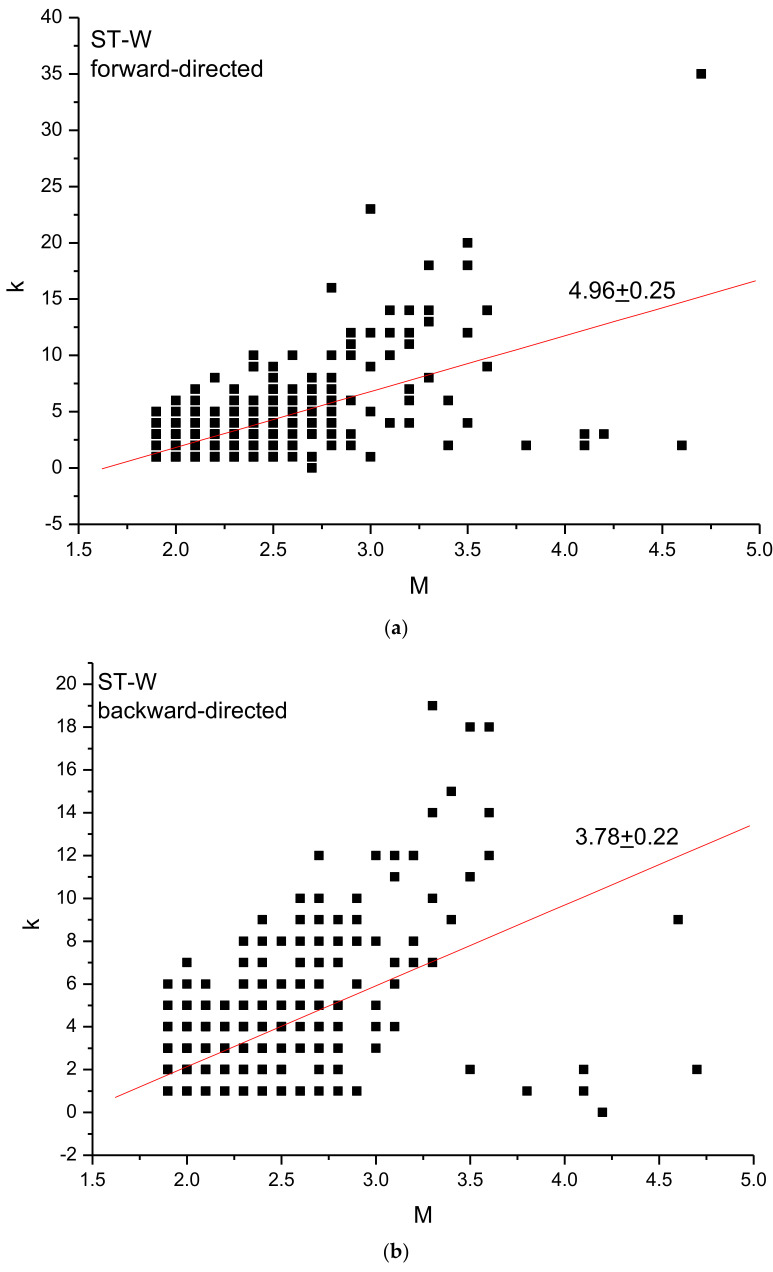

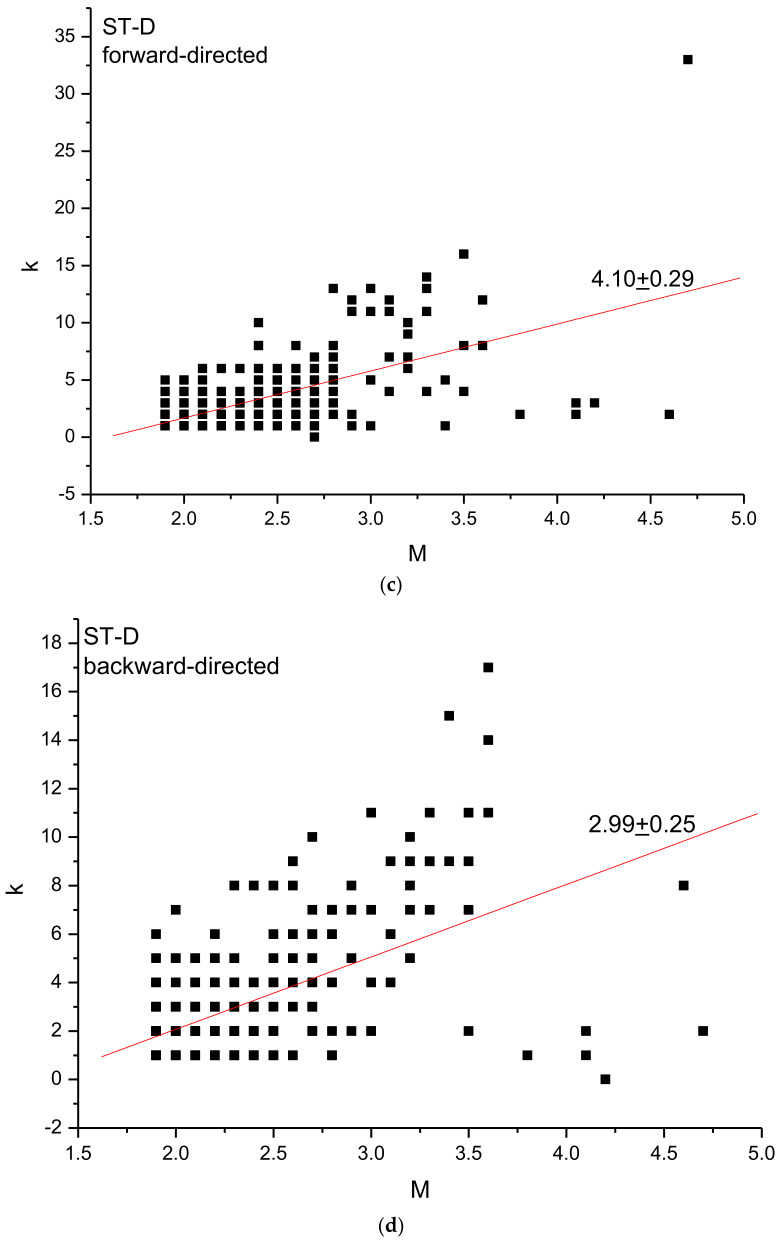
Relationship of the *b*-value with forward *k-M* slope (**a**,**c**) and with backward *k-M* slope (**b**,**d**) for ST2 catalogues.

Figure 6 shows the forward and backward connectivity degree versus magnitude for the whole and declustered ST2 seismicity. The forward and backward *k–M* slope is 4.96 ± 0.25 and 3.78 ± 0.22 for the whole seismicity and 4.10 ± 0.29 and 2.99 ± 0.25 for the declustered catalogue. The difference and ratio between the forward and backward *k–M* slopes can be considered as measure of the “anisotropy” of the catalogue [20]; thus the larger these quantities, more anisotropic the catalogue. In [20], these two quantities were used to characterize the “anisotropising” effect of the aftershocks of tectonic seismicity. In our case, the difference is 1.18 (ST-W) and 1.11 (ST-D) and the ratio is 1.31 (ST2-W) and 1.37 (ST-D); thus, both the whole and declustered catalogues have rather similar values of difference and ratio between the forward and backward *k–M* slopes, indicating that declustering does not change significantly the isotropic characteristics of the catalogues.

## 7. Discussion

The relationship between the *b*-value and the *k–M* slope has only very recently emerged as a challenging topic. Experimental works have shown that this relationship is positive, indicating an increase in the *k–M* slope with an increase in the *b*-value. The results obtained in the present study confirm the positive correlation between these two parameters.

**Figure 7:**
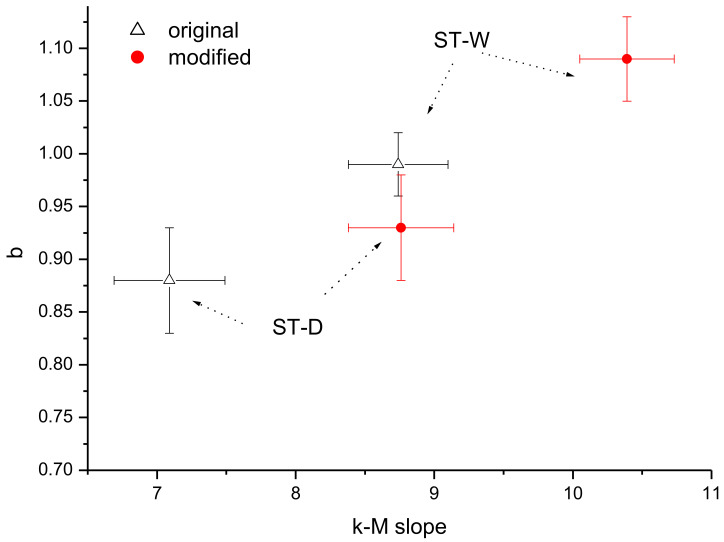
Relationship between the *b*-value and the *k–M* slope for the original (white triangle) and modified (red circle) ST2 catalogues.

The larger events generally behave as a “hub” of the seismic series, “attracting” more events than the smaller ones, and this is revealed by their larger connectivity degree. Such “hub” characterization features the larger earthquake if they are preceded and followed by events with significantly smaller magnitude than that of the “hub”, and this happens if the “hub” is “isolated”. If two or more events with relatively large magnitude follow each other, the “hub” characteristics cannot be maintained at least for one of them, leading to a connectivity degree that is lower than that of an earthquake with same magnitude but appearing “isolated”. In our case, the first five events of both the whole and declustered catalogues have magnitude ranging from 3.5 to 4.2 (included in the blue ellipse of Figure 5); although their magnitude is relatively large, their connectivity degree is rather small compared with events of similar magnitude. Their ordering is the following: 4.2 (3), 4.1 (3), 4.1 (5), 3.8 (3) and 3.5 (6) (the connectivity degree is indicated in brackets); as it can be clearly seen, the connectivity of the first earthquake with magnitude 4.2 is impeded by the second event with magnitude 4.1, whose connectivity is, in turn, impeded by the third earthquake with magnitude 4.1, and so on. Could this particular pattern, which does not depict these first five events as “isolated hubs”, have any effect on the relationship between the b-value and the k–M slope? In order to answer to this question, we removed these first five events from the ST2 catalogues and re-applied the VG method; the modified whole and declustered catalogues have, respectively, a *b*-value of 1.09 ± 0.04 and 0.93 ± 0.05 and a *k–M* slope of 10.39 ± 0.34 and 8.76 ± 0.38 (Figure 7).

The first five relatively large events therefore lead to a lower *b*-value and a lower *k–M* slope. Removing these events, in fact, changes the proportion between small and large events in favour of the first ones, increasing the proportion of the small events that causes an increase of the slope of the semi-log frequency–magnitude distribution (the *b*-value). The removal of these first five events also affects the topology of the seismicity with increased *k–M* slope.

**Figure 8:**
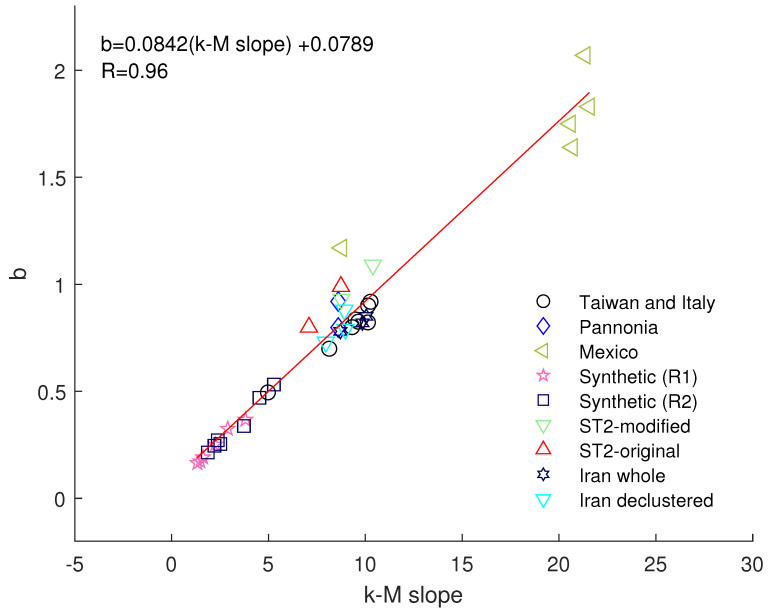
Relationship between the *b*-value and *k–M* slope for different seismic catalogues: ST2, Taiwan and Italy [20], Pannonia [17], Iran [18], Mexico [11], synthetic seismicity [21].

Although the mechanisms of generation of reservoir-triggered seismicity are different from those characterizing tectonic seismicity, the positive relationship between the *b*-value and the *k–M* slope still holds, strengthening the idea of its universality. To better emphasize this aspect, Figure 8 shows the results obtained in the present paper, including others obtained in previous studies. Fitting all the data by a regression line, we find that the relationship between the *b*-value and the *k–M* slope is well represented by the linear law *b* = 0.0842 · (*k–M* slope) + 0.0789 with a *R* factor of 0.96. An *R* factor near unity strengthens the identification of a strong linear correlation between the *b*-value and the *k–M* slope.

Khoshnevis et al. [18] found *b* ~ 0.084 · (*k–M* slope) and *b* ~ 0.085 · (*k–M* slope) for datasets including Iran whole and declustered catalogues, respectively. Azizzadeh-Roodpish and Cramer [19] found *b* ~ 0.0742 · (*k–M* slope). Telesca et al. [20] proposed *b* ~ 0.0749 · (*k–M* slope). The results found in [20] are rather different from that found by [18] because they represent the relationship obtained only for selected seismic areas (Taiwan and Italy). The higher similarity between Khoshnevis et al.’s relationship and that found in this study depends on also including the synthetic seismicity obtained by laboratory experiments (R1 and R5) and the Mexican seismicity that is characterized by a larger *b*-value.

The relationship shown in Figure 8 shows also that the data analysed so far seem clustered in three clusters discriminated by the *b*-value: smaller than ~0.6, intermediate between ~0.6 and ~1.3 and larger than ~1.6.

Including the ST2 data has strengthened the correlation between the *b*-value and the *k–M* slope and contributed to making their relationship more robust.

## 8. Conclusions

In this study, we analysed the seismicity triggered by Song Tranh 2 hydropower (Vietnam) by means of the visibility graph method. Our aim was to investigate the relationship between the Gutenberg–Richter *b*-value and the *k–M* slope of seismicity. Our results suggest a close agreement with similar relationships found in previous studies focused only on tectonic seismicity. The seismic clusters, for which the role was analysed by using the directed visibility graph, did not influence the isotropic behaviour of the catalogue. The results obtained for the reservoir-triggered seismicity of Song Tranh 2 area strengthen the relationship between the *b*-value and the *k–M* slope, although more case studies would be necessary to establish its universal character as robustly as possible.

## Data Availability

Not applicable.
