# Correction: Telesca et al. Visibility Graph Analysis of Reservoir-Triggered Seismicity: The Case of Song Tranh 2 Hydropower, Vietnam. *Entropy* 2022, *24*, 1620

**DOI:** 10.3390/e26121003

**Published:** 2024-11-22

**Authors:** Luciano Telesca, Anh Tuan Thai, Michele Lovallo, Dinh Trong Cao

**Affiliations:** 1 Institute of Methodologies for Environmental Analysis, National Research Council, 85050 Tito, Italy; 2 Institute of Geophysics, Vietnam Academy of Science and Technology, Hanoi 100000, Vietnam; tatuan@igp.vast.vn (A.T.T.); trongcd3284@gmail.com (D.T.C.); 3 Agenzia Regionale per la Protezione dell’Ambiente della Basilicata (ARPAB), 85100 Potenza, Italy; michele.lovallo@arpab.it

There was an error in the original publication [1]. The linear law *b* = 0.084 · (*k*–*M* slope) + 0.003 with an *R* factor of 0.98, referred to in Section 7, should be corrected as follows:

*b* = 0.0842 · (*k*–*M* slope) + 0.0789 with an *R* factor of 0.96.

Moreover, Figure 8 has been revised accordingly to the following:

The authors state that the scientific conclusions are unaffected. This correction was approved by the Academic Editor. The original publication has also been updated.

## Figures and Tables

**Figure 8:**
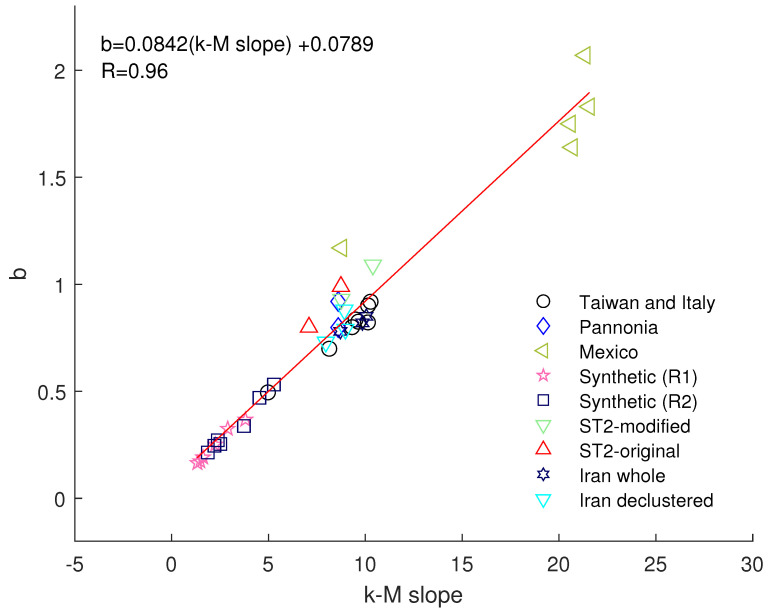
Relationship between the *b*-value and *k*–*M* slope for different seismic catalogues: ST2, Taiwan and Italy [20], Pannonia [17], Iran [18], Mexico [11], synthetic seismicity [21].
